# Evidence and clinical implications of target antigen changes and reassessment after ADC therapy in solid tumors

**DOI:** 10.3389/fonc.2026.2000729

**Published:** 2026-10-19

**Authors:** Zhao Wang, Yunze Shi, Songjiang Liu

**Affiliations:** 1 Graduate School, Heilongjiang University of Chinese Medicine, Harbin, China; 2 The First Affiliated Hospital of Heilongjiang University of Chinese Medicine, Harbin, China

**Keywords:** acquired resistance, antibody–drug conjugates, reassessment, sequential therapy, target antigen, tumor heterogeneity

## Abstract

There is growing interest in whether target antigens change after antibody–drug conjugate (ADC) therapy and how well previous test results reflect the current tumor state. Several studies have reported decreased, unchanged, or increased antigen expression after treatment. These findings are often influenced by the timing and site of sampling and the variables measured, and their meaning needs to be interpreted in the context of the specific drug and treatment setting. This review examines the evidence for post-treatment target antigen reassessment using HER2, FRα, TROP2, Nectin-4, HER3, and CLDN18.2 as representative targets. It draws on studies of patient specimens and ADC mechanisms of action to discuss the relationships between antigen changes, drug sensitivity, biomarker classification, and subsequent treatment selection. Reassessment after treatment can update previous test information and provide clues to ADC response and resistance. Reduced antigen expression may affect drug recognition and delivery. Disease progression despite retained expression suggests that antibody binding, intracellular processing, and payload sensitivity also need to be considered. For treatments with antigen expression requirements, reassessment can provide updated biomarker information.

## 1 Introduction

Antibody–drug conjugates (ADCs) use antibodies to bind tumor-associated antigens and deliver cytotoxic payloads to tumor cells (1, 2). This process is influenced by several factors, including target antigen distribution, the efficiency of drug entry into tumor cells, and payload activity. Although target antigens are central to drug design, biomarker requirements for patient selection differ among ADCs (2). As some ADCs move into earlier treatment settings, such as enfortumab vedotin (EV) combined with pembrolizumab as first-line treatment for advanced urothelial carcinoma, post-treatment resampling and sequential therapy have become more pressing issues (3).

A previous positive result documents the status of the sampled site at that time. Paired tissue analyses may show a decrease in HER2 after trastuzumab deruxtecan (T-DXd) treatment, whereas EV-resistant specimens may retain Nectin-4 (4, 5). The former suggests that previous test results may no longer accurately reflect the target antigen status of the current lesion; the latter indicates that treatment failure is not necessarily caused by target antigen loss. Previous reviews of HER2 loss have mainly addressed changes in tumor biology and differences arising from testing methods (6). As ADC use has expanded, recent expert discussions have also considered patient selection, specimen heterogeneity, and sequential therapy (2). We therefore searched PubMed, Europe PMC, and Embase using ADC drug and target names in combination with related terms, including expression, resistance, biopsy, and paired, and traced the references of relevant studies. Searches and supplementary updates covered literature through October 7, 2026. We focused on studies of patient specimens obtained after ADC treatment, comparing sampling time points, sites, and testing methods. We also considered mechanistic and clinical studies to discuss how antigen changes relate to drug response, resistance, and expression classification. Post-treatment retesting can provide additional information on the target in the current lesion and can be used to investigate whether antigen changes are associated with the efficacy of subsequent ADCs, and whether these results can help guide subsequent treatment selection.

## 2 Evidence supporting target antigen reassessment after ADC treatment

### 2.1 Comparability of pretreatment and post-treatment specimens

A prerequisite for determining whether an antigen has changed after treatment is that the tissues used for the two tests are comparable. Sampling studies in ovarian cancer and comparisons of primary and metastatic lesions in bladder cancer have shown that ADC target expression may differ between lesions (7, 8). Thus, when the baseline specimen is taken from the primary tumor and the specimen at progression is taken from a metastasis, the observed change may be related to treatment but may also be influenced by the sampling site. Testing methods and pathological interpretation can also introduce differences. Some interobserver disagreement exists at the boundary between HER2 immunohistochemistry (IHC) scores of 0 and 1 +. In cases with low expression, the staining system, cell proportions near scoring cutoffs, and background staining may all affect the final classification (9–11). Cursano et al. also obtained different expression classifications when testing the same set of 26 triple-negative breast cancer (TNBC) specimens with three TROP2 IHC systems (12). This suggests that some differences in scores may arise from the testing method itself and do not necessarily represent a true post-treatment change in the antigen. Similar findings have been reported for CLDN18.2: in paired primary and metastatic cases, changing the scoring rules altered both the positivity rate and the rate of classification discordance within the same set of cases (13). Comparisons of pretreatment and post-treatment antigen results should therefore be interpreted in relation to the sampled lesions, testing systems, and scoring rules.

Artificial intelligence (AI)-assisted pathology offers a way to reduce differences between readers. In one multireader HER2 study, AI assistance increased interobserver agreement from 69.8% to 87.4% for 58 HER2 0/1+ specimens with high-confidence expert reference scores (14). A second study, which included 853 slides from 581 patients, reported an increase in overall agreement from 79.8% to 88.6% with AI assistance (15). AI-assisted analysis has also been used to quantify staining for other targets. For example, a study in non-small-cell lung cancer measured membranous and cytoplasmic TROP2 staining separately and compared the results with pathologists’ assessments (16). For post-treatment reassessment, these methods may help score pretreatment and post-treatment specimens consistently and reduce the influence of reader differences on the interpretation of antigen changes. Their performance in paired specimens collected before and after ADC treatment still needs to be tested. Pathologist review and consistent tissue processing remain necessary, and differences caused by sampling different lesions need to be considered separately.

### 2.2 Expression changes and treatment stage

Studies of patient tissues have documented reductions in target antigens after ADC treatment, with decreases in HER2 after T-DXd and in folate receptor α (FRα) after mirvetuximab soravtansine (MIRV) providing representative examples (4, 17). However, specimens collected at different time points do not necessarily reflect the same biological state. Early on-treatment biopsies mainly reflect recent drug effects, whereas specimens collected at progression are also influenced by continued treatment exposure and tumor evolution. These two types of material should therefore not simply be grouped together as “post-treatment” specimens.

Target antigen expression does not invariably decrease after ADC treatment. In a small number of paired tissues obtained before and after sacituzumab govitecan (SG), TROP2 expression increased in some cases and decreased in others, with no statistically significant difference in the paired comparison. Some resistant tissues obtained after EV retained or showed increased Nectin-4 expression (5, 18). These findings indicate that antigen expression alone should not be the sole focus when resistance develops. Antibody binding, intracellular processing, and payload activity may also change, and different mechanisms may coexist.

Target antigen changes have been detected approximately 3 weeks after a single dose, during treatment cycles 2–3, and after completion of multiple treatment cycles; the sampling times are listed in **Table 1** (19–22). These observations establish when a change was detected, rather than when it began. For example, some patients receiving HER3-DXd continued to benefit after an early decrease in HER3 (20), showing that an early decrease in expression does not necessarily indicate resistance. Comparing how quickly different targets change will require consistent sampling schedules and serial observations.

**Table 1:** Target antigen expression and localization in patient specimens before and after ADC treatment.

Target and ADC	Assays in patient samples	Study and analyzable samples	Sampling and main findings	Implications for target antigen reassessment
HER2 T-DM1 in combination with pertuzumab	HER2 tissue IHC	Li et al. Breast cancer in the neoadjuvant setting; 49 patients with paired HER2 IHC samples (19).	Pretreatment biopsies were compared with residual tumors resected within 42 days of the last dose, after 6 cycles of T-DM1 plus pertuzumab given every 3 weeks. HER2 protein expression decreased in 26 of 49 paired specimens.	Residual tissue at surgery records HER2 status in tumor cells surviving treatment and can reveal expression that differs from pretreatment findings.
HER2 T-DM1	HER2 tissue IHC	Schwarz et al. Breast cancer; paired pretreatment and post-progression tissue from 11 patients (44).	HER2 protein expression decreased in 7 of 11 patients when tissue obtained before T-DM1 was compared with tissue collected after progression. Patients had previously received anti-HER2 therapy.	HER2 expression in some lesions was lower at progression than before treatment. Retesting can identify this change and provide tissue evidence for investigating the relationship between target downregulation and resistance.
HER2 T-DM1	HER2 tissue IHC	Giordani et al. Breast cancer; paired pre- and post-treatment tissue from 3 patients (45).	Pretreatment samples were collected less than 1 year before T-DM1 initiation; progression samples were obtained within 1 week after the last dose. IHC scores increased from 2+ to 3+, 1+ to 2+, and 2+ to 3+ in the 3 pairs. Sampling sites were not identical in all pairs.	These paired samples document increased HER2 scores at progression on T-DM1. Post-treatment antigen changes can also include increased expression; pretreatment and post-treatment sampling sites should be recorded when making comparisons.
HER2 T-DM1	HER2 tissue IHC	Lee et al. Breast cancer; paired tissue obtained before T-DM1 and after resistance developed in 5 patients (27).	HER2 expression decreased in 3 of 5 patients with paired pretreatment and resistant tissue.	These findings provide further examples of HER2 downregulation after T-DM1 resistance, indicating that previous expression results may not fully reflect target status at progression.
HER2 T-DM1	HER2 IHC (4B5; endometrial cancer scoring criteria), with FISH	Chui et al.; recurrent high-grade endometrial cancer. Pre- and post-treatment data for 3 T-DM1-treated patients could be separately extracted from a cohort receiving different treatments (58).	Pretreatment and post-treatment recurrent/progressive samples: 1 case, 3+→1+; 2 cases, 3+→3+. Sampling sites differed, and individual sampling intervals were not reported.	These cases document both decreased and retained HER2 expression after T-DM1 in endometrial cancer. Reduced protein expression can coexist with retained amplification.
HER2 T-DXd	HER2 tissue IHC	DAISY Breast cancer; 186 patients enrolled; paired expression data before treatment and at resistance were available for 20 patients (42).	Patients were classified at baseline as HER2-positive, HER2-low, or IHC 0. HER2 expression decreased in 13 of 20 analyzable baseline–resistance tissue pairs.	Retesting can identify lower HER2 expression at resistance than before treatment in some patients, updating target information for the current lesion. The decrease was assessed in 20 patients with analyzable paired samples.
HER2 T-DXd	HER2 tissue IHC	Gouda et al. Breast cancer; paired tissue from 41 patients, including 34 with HER2 expression in the most recent pretreatment sample (43).	The median duration of T-DXd treatment was 197 days. Pretreatment biopsies were obtained a median of 322 days before the first dose. Biopsies after exposure were obtained a median of 45 days after the last dose (range, −13 to 700 days), including samples collected during treatment and after discontinuation. Of 34 patients with HER2 expression at the most recent baseline assessment, 11 were IHC 0 after exposure.	Retesting can identify changes in prior HER2 expression categories after T-DXd exposure and update expression information for current tissue. Sampling times varied widely; differences should be interpreted in relation to sampling intervals and intervening treatment.
HER2 T-DXd	Clinical HER2 IHC; additional tissue immunofluorescence	Chen et al. Breast cancer; paired clinical HER2 IHC results from 102 patients (4).	Pretreatment tissue obtained closest to T-DXd initiation was compared with post-progression tissue. HER2 IHC scores decreased in 50/102 patients, including 26/102 who became IHC 0. Separate immunofluorescence analyses also supported decreased expression.	This larger paired-tissue series further shows that HER2 status at progression can differ from pretreatment status. Retesting can identify these changes and update expression information for current lesions.
FRα MIRV	FRα tissue IHC FOLR1 2.1; BenchMark XT Proportion of cells with ≥2+ membrane staining	Martin et al. Ovarian cancer; 27 patients treated; early paired samples from 17 patients (63).	Sampling before treatment and on cycle 2, day 8. Eligibility required ≥25% of cells with ≥2+ staining and was not restricted to high expression. Expression was retained in most early paired samples and decreased in some low-expression cases.	Enrollment was not restricted to patients with high FRα expression, and retesting occurred early during treatment. Expression was retained in most samples at this stage. This study and progression studies document target status at different stages of MIRV treatment.
FRα MIRV	FRα tissue IHC FOLR1 2.1; BenchMark Ultra Membranous H-score	Johannet et al. Ovarian cancer; 170 patients treated; paired samples at progression from 12 patients (17).	Patients with high FOLR1 expression received standard-of-care MIRV monotherapy. Post-treatment tissue was available for 12/170 patients. Expression decreased in 10/12; median H-score, 245→130; P = 0.005.	Patients with previously high FRα expression can show decreased expression at progression; retesting updates expression results for the current lesion. The proportion with decreased expression was calculated among the 12 patients with paired tissue. Whether expression still meets the PS2+ high-expression criterion requires PS2+ assessment.
FRα MIRV MORAb-202	FRα tissue IHC; PS2+ expression categories	Lee et al.; paired tissue before and after ADC treatment in ovarian cancer. The number of patients with paired samples was not reported in the abstract. Published article abstract and ASCO meeting report (64, 65).	Before and after exposure to the corresponding ADCs; FOLR1 expression tended to decrease.	These findings add evidence on antigen changes after treatment with different FRα-directed ADCs.
TROP2 SG	TROP2 tissue IHC; membranous and cytoplasmic staining distribution	Coates et al. Triple-negative breast cancer; protein localization was compared in 1 patient from an autopsy series of 3 patients (29).	The pretreatment primary tumor showed prominent membrane staining. At autopsy after progression on SG, the hilar lymph node lesion showed predominantly cytoplasmic staining, whereas the brain metastasis retained membrane staining. The interval from progression to autopsy was less than 4 months, with brief intervening chemotherapy.	TROP2 can remain detectable in resistant lesions while its localization differs. Comparing membranous and cytoplasmic distribution adds target information beyond staining intensity. Lesion sites also differed in this case.
TROP2 SG	TROP2 tissue IHC; H-score	Weich et al. Breast cancer; 76 patients and 229 samples overall. The paired SG analysis included 13 patients, with 37 pretreatment and 13 post-treatment samples (18).	The primary analysis compared each patient’s mean H-score within the pretreatment and post-treatment time windows. Sampling was not standardized to progression. Expression decreased in 6 patients and increased in 7; mean H-score, 227.0→202.9; Wilcoxon P = 0.542. Sensitivity analysis comparing the last pretreatment and first post-treatment samples: 6 decreases, 3 increases, and 4 unchanged.	TROP2 changes after SG vary between patients: individual increases and decreases can occur despite a nonsignificant overall comparison. Using time-window means or single paired samples also changes the classification for some patients, showing that reassessment results depend on sampling and the comparison method.
TROP2 SG	Protein quantification by mass spectrometry (mPROBE); qualitative multiplex immunofluorescence	Mai et al.; 2026 ASCO meeting report. Paired tissue before and after SG from 14 patients, analyzable by mass spectrometry (70).	Before and after SG treatment. TROP2 changes ranged from −96% to +400% (median, −18%); the group comparison showed no significant overall change after adjustment.	TROP2 changes after SG varied considerably between patients. Mass spectrometry provides protein quantification that complements IHC.
TROP2 SG	Multispectral CTC immunofluorescence; categories based on single-cell expression intensity	Mishra et al.; metastatic breast cancer. The SG group included 13 patients, of whom 6 had paired CTC samples at baseline and progression (71).	TROP2 expression distributions showed no marked change in 5 patients. In 1 other patient, only 1 CTC with higher TROP2 expression was detected at progression.	Serial blood sampling can document antigen status on CTCs at progression, adding patient-level protein evidence beyond tissue biopsy. Expression distributions should be compared in light of the number of CTCs actually detected.
Nectin-4 EV	Nectin-4 tissue IHC; membranous H-score	Chang et al. Urothelial carcinoma; paired pre- and post-treatment samples from 17 patients (5).	Comparison of tissue obtained before EV and at resistance showed retained Nectin-4 expression in most resistant tumors; expression increased in 9 of 17 patients.	Nectin-4 can be retained or increased at EV resistance. Paired patient tissue can therefore distinguish target retention from loss, providing a basis for investigating the causes of resistance.
Nectin-4 EV	Nectin-4 tissue IHC; membranous H-score	Wakamiya et al. Renal pelvic urothelial carcinoma; 1 tissue pair obtained before treatment and at local progression (73).	Local progression occurred after cycle 11 of EV. Tissue resected after cycle 16 showed a decrease in Nectin-4 H-score from 200 to 5. Platinum-based chemotherapy and pembrolizumab had also been administered between the baseline biopsy and EV treatment.	This case documents marked Nectin-4 downregulation at progression on EV. Together with cohorts showing retained expression, it illustrates variation in target changes. The difference between samples must be interpreted in the context of all intervening treatments.
Nectin-4 EV + pembrolizumab	Tissue IHC; separate membranous and cytoplasmic H-scores; membranous negativity defined as <15	Sternschuss et al. Urothelial carcinoma; 46 patients had paired tissue, with 40 pairs analyzable by IHC (74).	Before and after EV combination therapy: median membranous H-score, 195→200 (P = 0.5); cytoplasmic H-score, 0→0 (P>0.9). Moderate/strong membrane expression persisted after treatment in 29/40 patients. Among 5 patients with post-treatment membranous negativity, 2 were already negative and 3 had weak expression at baseline.	Most patients retained membranous Nectin-4 after EV combination therapy. A negative post-treatment classification does not necessarily mean newly acquired complete loss; baseline levels also matter. The report distinguished membranous from cytoplasmic changes.
HER3 HER3-DXd	HER3 tissue IHC; proportion of cells with positive membrane staining	TOT-HER3 Part A Early breast cancer; 78 patients treated; paired HER3 IHC samples from 54 patients (21).	Previously untreated HR-positive/HER2-negative breast cancer, sampled before and 21 ± 3 days after a single dose. The median change in the proportion of cells with positive HER3 membrane staining was −5 percentage points.	Changes in the proportion of HER3-positive cells were detectable after a single dose. Early samples record target status during the period of recent drug exposure and provide information distinct from samples obtained at progression.
HER3 HER3-DXd	Central HER3 IHC; proportion of cells with positive membrane staining	Krop et al. Breast cancer; U31402-A-J101. A total of 182 patients were treated; early paired HER3 IHC samples were available for 15 patients (20).	In HR-positive/HER2-negative breast cancer, comparison of baseline biopsies with those obtained on cycle 2, day 3 or cycle 3, day 3 showed an overall decrease in the proportion of tumor cells with positive HER3 membrane staining. Some patients continued to derive treatment benefit after expression decreased.	Some patients continued to benefit from treatment despite an early decrease in the proportion of HER3-positive cells. Expression can therefore decrease while the drug remains active, and its meaning should be considered alongside subsequent tumor response.
HER3 HER3-DXd	Tissue IMC; HER3 protein and proportion of positive cells	ICARUS-BREAST01 Breast cancer; 99 patients treated; early paired IMC samples from 39 patients (36).	IMC samples included 61 at baseline and 57 on treatment, with 39 pairs available for comparison. On-treatment sampling was scheduled for cycle 1, day 3; cycle 1, day 19; or cycle 2, day 3. The pooled analysis showed a greater decrease in the proportion of HER3-positive cells in responders.	The greater early decrease in the proportion of HER3-positive cells among responders indicates that this change can accompany treatment response. Its meaning should be distinguished from antigen downregulation at progression.
CLDN18.2 AZD0901 (CMG901)	CLDN18.2 tissue IHC; SP455; blinded scoring	Terán Brage et al. CLARITY-PT-01; advanced/metastatic gastric cancer. Paired baseline and cycle 2 samples were collected from 22 patients; 15 pairs were analyzable by IHC (22).	Baseline versus cycle 2 (the abstract did not report the exact sampling day): 7 pairs were unchanged, 6 decreased, 1 increased, and 1 was negative at both time points. Among those with decreased expression, 1 fell below the threshold of staining in 25% of tumor cells.	Antigen retention or downregulation was detectable early during treatment, with most samples remaining positive. This meeting report adds direct patient IHC evidence after a CLDN18.2-directed ADC and describes early changes.

Summary of 22 study reports that directly assessed target antigen protein expression or localization in patient specimens. Studies reporting only gene or RNA changes are discussed in the main text.

IHC, immunohistochemistry; FISH, fluorescence *in situ* hybridization; IMC, imaging mass cytometry; CTC, circulating tumor cell. The H-score combines staining intensity and the proportion of cells, whereas PS2+ measures the proportion of cells meeting the specified membrane staining intensity. The table includes only antigen protein findings in patient specimens; findings on drug distribution, gene and RNA testing, and functional models are discussed in the main text. “Before and after” or “paired” means that samples were available from the same patient before and after ADC exposure; it does not guarantee that they were obtained from the same lesion or that the patient received only that ADC between sampling time points.

### 2.3 Antigen binding and intracellular drug activity

IHC mainly reflects antigen distribution and staining intensity and cannot directly determine whether the therapeutic antibody binds its target sufficiently. HER2 studies combining post-treatment patient tissue findings with functional experiments have shown that both reduced HER2 expression and specific changes at the antibody-binding site can weaken T-DXd activity (4). Therefore, even when the antigen remains detectable, the ability of the therapeutic antibody to bind the target effectively must still be considered.

The efficiency of ADC entry into cells may differ even when antigen expression is similar. In resistant models, Wang et al. found that some cells with high Nectin-4 expression reduced intracellular drug accumulation by decreasing endocytosis and increasing efflux. Paired spatial transcriptomic analyses of patient samples also identified a tumor state with higher NECTIN4 RNA levels and downregulated endocytosis-related pathways (23). After entering cells, ADCs must undergo processes including lysosomal processing and active payload release. In some models resistant to trastuzumab emtansine (T-DM1), HER2 expression and early endocytosis were retained, but lysosomal acidification and proteolysis were impaired (24). Cell experiments by Tomabechi et al. further showed that, after T-DM1 is degraded in lysosomes, its active catabolite can be transported by SLC46A3; inhibition of this process reduces the drug’s cytotoxicity against tumor cells (25). These studies show that antigen retention does not guarantee effective drug uptake and completion of intracellular processing.

Drug efflux may also affect intracellular drug accumulation. In an analysis of tissues from patients with breast cancer, Sledge et al. found that RNA levels of the drug efflux-related gene ABCC1 were higher in the group sampled after T-DXd initiation than in the pretreatment group. Higher pretreatment ABCC1 levels were also associated with shorter overall survival (26). This study compared two groups of different patients, and its findings provide clues from patient tissues for investigating efflux-related resistance.

Tumor sensitivity to the payload also affects ADC activity. RNA analysis of 3 tissue pairs obtained before and after T-DXd treatment showed enrichment of DNA repair-related gene sets at progression (27). Alterations in topoisomerase I (TOP1) have been observed in patient data and functional experiments (28). In a case of acquired SG resistance, different metastatic clones showed alterations involving either the antigen or the payload target (29). In SG-resistant and T-DXd-resistant breast cancer models, Rampa et al. observed cross-resistance to TOP1 inhibitor payloads, whereas experimental ADCs using microtubule inhibitor payloads retained antitumor activity in cells and xenografts (30). Even if a subsequent ADC targets a different antigen, pre-existing payload-related resistance may continue to affect treatment efficacy if the new ADC uses a similar type of payload. Analysis of post-treatment antigen changes should therefore also consider the antibody, linker, and payload of the specific ADC. The patient specimens described above provide clues to resistance, while functional experiments test whether these changes affect drug activity. Using the findings to select subsequent treatment requires linking the test results to patients’ outcomes on that treatment. **Figure 1** summarizes antigen differences before and after treatment and the related processes of drug action.

**Figure 1:**
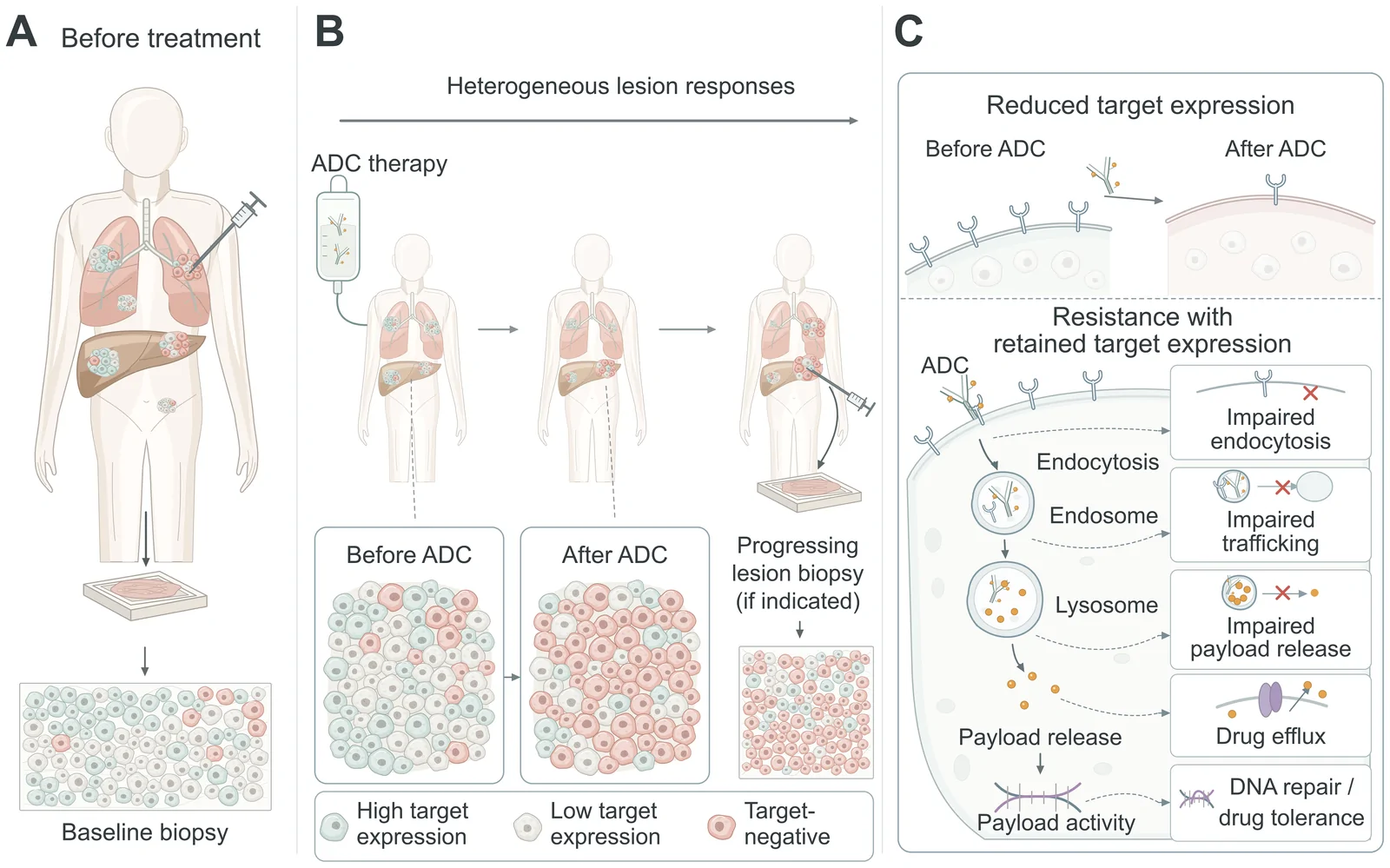
Biological rationale for target antigen reassessment after ADC treatment. **(A)** Before treatment, antigen expression may vary between lesions and within the same lesion. **(B)** Clonal composition and lesion responses may change after ADC exposure. The biopsy of a progressing lesion illustrates a source of post-treatment tissue and does not represent a uniform sampling requirement. **(C)** Resistance may be accompanied by reduced antigen expression, or the antigen may be retained while endocytosis, trafficking, payload release, or payload activity is impaired. These mechanisms may coexist.

## 3 Post-treatment evidence and clinical interpretation for representative targets

ADCs targeting HER2, FRα, TROP2, and Nectin-4 have been approved for the treatment of solid tumors (31–34), and ADCs targeting HER3 and CLDN18.2 have entered phase II or III clinical studies (35, 36). Based on the clinical progress of these drugs and the available patient specimen data, this section examines these six representative targets, focusing on changes in target antigen expression and localization after ADC treatment. For all six targets, patient specimen studies have compared target antigen protein expression or cellular localization before and after ADC treatment. These direct measurements are summarized in **Table 1**.

### 3.1 HER2

#### 3.1.1 Expression classification and subsequent treatment in breast cancer

Patient selection criteria are not identical across HER2-targeted ADCs. T-DM1 is used mainly for HER2-positive breast cancer (37, 38), whereas evidence for T-DXd has expanded to HER2-low and hormone receptor (HR)-positive HER2-ultralow populations (34, 39). In the assay validation study for DESTINY-Breast06, some samples historically recorded as IHC 0 were classified as HER2-low or HER2-ultralow by central testing (40). Because the two assessments may have used different specimens and reagents, this difference cannot be interpreted as a misclassification rate for the same slide or as a change in HER2 after ADC treatment. Tarantino et al. used quantitative immunofluorescence to analyze breast cancer tissues obtained from 51 patients before T-DXd treatment and found that higher HER2 levels were associated with longer overall survival (41). This finding provides a basis for investigating whether post-treatment changes in HER2 abundance are associated with the efficacy of subsequent ADCs.

Of the 20 evaluable specimen pairs in the DAISY resistance analysis, 13 showed decreased HER2 expression (42). Gouda et al. included 41 patients with metastatic breast cancer who had paired tissues obtained before and after T-DXd exposure. HER2 expression was present in the most recent pretreatment specimen in 34 patients; after exposure, 11 of these patients had IHC 0, and another 10 had a downward shift in HER2 classification according to the study definition (43). Samples were collected both during treatment and after discontinuation, so changes between the two assessments must be interpreted in relation to the sampling interval and intervening treatments. These studies show that the pretreatment HER2 classification may change after T-DXd exposure and that post-treatment tissue can provide additional information on expression in the current lesion. DAISY also provided information beyond antigen expression. Among 6 patients with resistance in the HER2-overexpressing cohort, intratumoral T-DXd remained detectable in 4 resistant specimens collected no more than 6 weeks after the last infusion (42). This finding shows that resistance developed in some lesions despite detectable T-DXd. This analysis involved 6 patients and should be interpreted separately from the HER2 expression analysis of 20 patients because the denominators differ. HER2 scoring and intratumoral drug detection therefore address different questions. Further studies could examine whether active payload can still be released and act within cells in lesions with decreased or retained HER2 expression.

HER2 protein expression and ERBB2 amplification do not always change in parallel after T-DM1 treatment, and signaling through related receptors may also change. Schwarz et al. observed decreased HER2 in 7 of 11 tissue pairs obtained before treatment and after progression, with increased phosphorylated HER3 in some cases (44). Changes in HER2 protein expression and ERBB2 amplification may also differ in residual tissues after neoadjuvant T-DM1 combined with pertuzumab (19). In the study by Giordani et al., DNA testing detected no ERBB2 amplification in any of 5 tissues obtained at progression on T-DM1, although 4 remained HER2 IHC 3 +. IHC scores increased in all 3 patients for whom paired pretreatment and post-treatment tissues were available (45). These results show that protein expression, gene amplification, and signaling through related receptors may change differently in post-treatment lesions. Separate measurements can provide information not captured by a single HER2 result. A decrease in the HER2 score does not necessarily mean that a patient no longer meets the expression criterion for a particular treatment. For example, a tumor can still be classified as HER2-positive if IHC decreases from 3+ to 2+ while *in situ* hybridization (ISH) remains positive. When IHC decreases from 1+ to 0, the presence or absence of membrane staining is needed to further distinguish HER2-ultralow expression (34). Even when retesting shows a change, whether subsequent treatment should be adjusted on that basis must still be considered in light of the clinical evidence for the specific treatment sequence.

Available evidence on sequential therapy shows that failure of one HER2-targeted ADC does not necessarily eliminate all opportunities for treatment directed at the same target. DESTINY-Breast02 demonstrated that T-DXd was superior to treatment of physician’s choice in HER2-positive advanced breast cancer after progression on T-DM1 (46). This sequence retains HER2 as the target but changes the payload class. In contrast, sequencing SG and T-DXd changes the target while retaining TOP1 inhibitor payloads. In relevant retrospective studies, progression-free survival or time to treatment failure was generally short with the second ADC, although outcomes varied considerably between patients (47, 48). These studies evaluated specific treatment sequences rather than comparing retesting and no-retesting strategies. An exploratory analysis of KATHERINE also showed that some patients with HER2-positive disease before treatment whose residual tumors became HER2-negative after non-ADC neoadjuvant therapy could still benefit from adjuvant T-DM1 (49). However, this finding applies to a specific adjuvant setting and cannot be directly extrapolated to sequential treatment after progression on an ADC in advanced disease. In breast cancer, reassessment first establishes the HER2 expression category of the current lesion after ADC exposure and identifies whether previous results still represent that lesion (42, 43). Whether a patient still meets a treatment’s expression requirement and whether that treatment will be effective are separate questions. Current studies support the use of retesting to update expression classification; they have not established that choosing the next ADC according to post-treatment HER2 changes improves outcomes.

#### 3.1.2 Reassessment in gastric and gastroesophageal junction adenocarcinoma

In gastric cancer, patient studies and the prescribing information support reconfirming HER2 after treatment with the unconjugated antibody trastuzumab and before the first T-DXd treatment. GASTHER3 included 48 patients with paired samples, of whom 14 no longer met the criteria for HER2 positivity after trastuzumab-containing treatment (50). DESTINY-Gastric02 required HER2 positivity to be reconfirmed using newly obtained material after progression on trastuzumab before enrollment (51), and the phase III randomized DESTINY-Gastric04 study used a similar requirement (52). The prescribing information also states that HER2 may be reassessed when a new specimen can be obtained (34). These data clarify the purpose of reconfirming HER2 after trastuzumab and before the first T-DXd treatment: to use a current specimen to determine whether the patient meets the expression criterion for this treatment. Kapil et al. performed computational pathology analysis of pre-T-DXd tissues from DESTINY-Gastric01 and found that HER2-positive cell density and a score reflecting their spatial distribution were associated with progression-free survival. This association was validated in 72 patients from DESTINY-Gastric02 (53). This suggests that, when studying post-treatment changes in HER2, the density and distribution of positive cells also merit attention alongside expression categories.

Providing additional evidence on resistance mechanisms, the spatial study of gastric cancer by Sheng et al. included paired tissues obtained before T-DXd treatment and after progression. The study included 30 patients, of whom 6 had received T-DXd; only 3 had these paired specimens (54). The study observed reduced human leukocyte antigen (HLA)-related transcription and changes in oxidative phosphorylation. These findings mainly reflect immune and metabolic changes at resistance and provide transcriptomic information beyond HER2 protein testing. The two types of reassessment in gastric cancer serve different purposes: reassessment after trastuzumab and before the first T-DXd treatment confirms the expression criterion, whereas patient specimens obtained after progression on T-DXd provide clues to resistance.

#### 3.1.3 Testing variables in non-small-cell lung cancer and other solid tumors

The basis for HER2 assessment for T-DXd differs across cancer types. In advanced non-small-cell lung cancer with an activating ERBB2 mutation, treatment selection is based primarily on the relevant gene mutation rather than the HER2-low classification used in breast cancer. If the relevant mutation is not detected in plasma, the prescribing information requires further testing of tumor tissue (34, 55). The tumor-agnostic solid tumor indication is based on IHC 3+ and the corresponding prior-treatment criteria (34). Although DESTINY-PanTumor02 enrolled patients with IHC 2+ or 3+ tumors (56), its enrollment criteria cannot be used directly to define the approved population.

In a patient with HER2-mutant lung cancer, the tumor shrank after 2 cycles of neoadjuvant T-DXd, and paired whole-genome sequencing showed a decrease in ERBB2 copy number from 13.34 to 2.10 (57). This single case documents an early genetic change during treatment. A decrease in gene copy number does not directly demonstrate a reduction in HER2 protein on the cell surface. The endometrial cancer study by Chui et al. provided pretreatment and post-treatment data for 3 patients treated with T-DM1. HER2 IHC decreased from 3+ to 1+ in 1 patient and remained 3+ in the other 2; HER2 amplification was retained in the case with decreased expression (58). Thus, the HER2 test results used to select T-DXd differ between ERBB2-mutant lung cancer and IHC 3+ solid tumors. Post-treatment retesting also needs to distinguish protein expression, gene amplification, and activating mutations: protein assays describe HER2 expression in the current specimen, whereas molecular assays identify genetic alterations. Identification of other actionable alterations forms part of a broader assessment of resistance.

### 3.2 FRα

MIRV use requires patients to meet both the relevant disease criteria and the criterion for high FRα expression. Its indications include platinum-resistant epithelial ovarian, fallopian tube, and primary peritoneal cancers. The randomized evidence from MIRASOL comes from patients who had received 1–3 prior lines of treatment and met the specified expression requirement (59). High FRα expression is assessed using PS2+, which measures the proportion of viable tumor cells with moderate or strong membrane staining; the cited cutoff is at least 75% (33, 60). FORWARD I and subsequent consensus recommendations show that scoring methods affect patient classification, so expression cutoffs must be interpreted in relation to the specific testing system (61, 62).

Martin et al. obtained 17 pairs of specimens before treatment and on cycle 2, day 8 from a cohort of 27 patients. FRα expression was retained in most cases early in treatment (63). In the retrospective cohort of 170 patients receiving MIRV monotherapy reported by Johannet et al., post-progression tissue was obtained from only 12 patients. Of these, 10 showed decreased FRα (FOLR1) protein expression, and the median H-score in paired samples decreased from 245 to 130 (P = 0.005) (17). The two studies differed in their patients and sampling time points and provided expression data from early treatment and progression, respectively. The decrease observed in 10/12 patients in the Johannet study describes only those 12 patients from whom post-progression tissue was obtained. Interpretation should also consider pretreatment and post-treatment sampling sites, prior therapies, and baseline tissue heterogeneity. Lee et al. also reported a trend toward decreased FOLR1 expression in paired tissues obtained after MIRV or MORAb-202 treatment in a study of multiple ADC targets in ovarian cancer (64, 65).

The H-score incorporates both staining intensity and cell proportion, whereas PS2+ measures the proportion of cells reaching the specified membrane staining intensity. The two scores measure different aspects of expression, so a decrease in H-score does not necessarily mean that a patient no longer meets the PS2+-defined expression criterion. A decrease in H-score after MIRV indicates a change in FRα expression, but whether it crosses the eligibility cutoff for another FRα-targeted treatment must still be assessed using the relevant testing criteria. The relationship with subsequent efficacy may also be influenced by resistance to DM4 and the payload characteristics of different ADCs. FRα reassessment therefore needs to assess both antigen abundance and the proportion of cells positive under the intended drug’s scoring criteria. This can establish whether reduced expression also changes eligibility under those criteria. Early retention and decreases at progression were observed in different patients and do not establish a common course of change for every patient (17, 63). After progression on MIRV, the available data mainly support updating FRα status. Benefit from a subsequent FRα-targeted ADC needs to be evaluated against actual treatment outcomes.

### 3.3 TROP2

SG and datopotamab deruxtecan (Dato-DXd) both target TROP2, but their currently approved uses do not specify a TROP2 IHC cutoff for routine patient selection. Each drug nevertheless has its own clinical and molecular criteria. For example, first-line SG plus pembrolizumab for TNBC requires a PD-L1 CPS ≥10, whereas the relevant lung cancer indication for Dato-DXd requires an EGFR mutation and prior EGFR-targeted therapy and platinum-based chemotherapy (32, 66). These criteria primarily define the treatment population; they do not establish whether post-treatment TROP2 changes can inform drug selection. ASCENT and TROPiCS-02 established the efficacy of SG in previously treated TNBC and HR-positive/HER2-negative advanced breast cancer, respectively (67, 68). An exploratory ASCENT analysis included 290 patients with TROP2 expression data. Outcomes with SG were numerically better than those with chemotherapy in the high-, medium-, and low-expression groups, although the low-expression group was small (69). A subsequent expression subgroup analysis showed a similar pattern, but the testing was exploratory and *post hoc*, and staining results were available for only a subset of randomized patients (68). These data mainly describe the relationship between pretreatment TROP2 expression and efficacy and have not established a reliable criterion for excluding patients from treatment.

In 13 patients who received SG, mean TROP2 H-scores within each patient’s pretreatment and post-treatment time windows were compared. Expression decreased in 6 patients and increased in 7, with no statistically significant difference in the paired Wilcoxon comparison (18). Sampling was not uniformly scheduled at disease progression, so these results mainly describe TROP2 expression changes within the observed time windows. In a report presented at ASCO 2026, Mai et al. analyzed paired tissue samples collected before and after SG from 14 patients. Mass spectrometry quantification showed TROP2 changes ranging from −96% to +400%, with a median change of −18%; the group analysis showed no significant overall change after correction for multiple testing (70). Serial blood sampling also provided antigen data at progression. Mishra et al. compared circulating tumor cells (CTCs) before SG and at progression in 6 patients. The distribution of TROP2 expression showed no clear change in 5 patients; in the remaining patient, only 1 CTC was detected at progression, and it had higher TROP2 expression (71).

In addition to TROP2 expression, Mai et al. measured TOP1, the target of the SG payload. Among 14 pairs of tissue samples collected before and after treatment, TOP1 protein decreased by more than 25% in 6 patients (70). Other patient studies have also observed TOP1 alterations and TROP2 changes in different tumor clones (28, 29), indicating that resistance may involve both the target antigen and tumor sensitivity to the payload. Post-treatment testing can therefore examine both aspects: TROP2 results describe target status, while TOP1 findings provide clues to payload sensitivity. Even when TROP2 is retained, payload-related resistance may affect the efficacy of the next ADC carrying a TOP1 inhibitor. At present, TROP2 retesting mainly helps investigate resistance; these studies have not established an expression cutoff for selecting or excluding a subsequent ADC (18, 28, 29, 70, 71).

### 3.4 Nectin-4

Neither EV monotherapy nor EV combination therapy for advanced urothelial carcinoma uses a Nectin-4 IHC cutoff for patient selection (31, 72). Klümper et al. analyzed available tumor tissue from 47 patients who received EV and found that patients with absent or low membrane Nectin-4 expression had shorter progression-free survival than those with moderate or high expression (8). This study examined the relationship between antigen status before EV and efficacy, providing a basis for further research on post-treatment expression changes.

Protein measurements before and after EV show that resistance is not necessarily accompanied by Nectin-4 loss. Chang et al. analyzed paired tissue samples collected before and after EV from 17 patients. Most resistant tumors retained Nectin-4 expression, and expression increased after treatment in 9 patients (5). In another case, a locally progressing lesion showed almost complete loss of Nectin-4 expression, with the H-score decreasing from 200 to 5. The patient had also received platinum-based treatment and immunotherapy between the two sampling time points, so this finding describes target status after exposure to multiple treatments (73). In patients treated with EV plus pembrolizumab, a conference report by Sternschuss et al. analyzed 40 tissue pairs collected before and after treatment. Moderate or strong membrane expression was retained after treatment in 29 patients, and neither membrane nor cytoplasmic H-scores showed a significant overall difference (74).

Molecular testing of patient tissue and functional models provide further explanations for resistance. Wang et al. compared tissue from the same lesion before EV plus pembrolizumab and at progression in 6 patients. They found an overall increase in NECTIN4 RNA levels, together with enrichment of a tumor state characterized by high AKR1C1 and ELF3 expression (23). The measured variable here was target-related RNA; further analyses of protein expression and drug uptake mainly came from models. To investigate Nectin-4 retained after EV resistance, Chang et al. studied Nectin-4-targeted chimeric antigen receptor T cells (CAR-T cells) in models, suggesting that this target may still be used through other approaches to kill resistant tumor cells (5). Another retrospective study of 20 patients treated with EV analyzed ATP-binding cassette (ABC) transporter expression in available primary tumor tissue and found that high expression was associated with shorter progression-free survival and poorer response (75). This analysis examined the relationship between expression and efficacy, providing clues for research on drug efflux.

In its discussion of treatment after progression on EV, the 2026 European Association of Urology (EAU) guidelines note that evidence for some subsequent regimens remains limited in this treatment sequence (76). Prior treatment and other molecular information also affect subsequent choices. When Nectin-4 is retained at progression on EV, retesting establishes that the target remains present in the sampled lesion, directing attention to drug uptake, payload activity, and other potential causes of resistance. These findings do not yet establish Nectin-4 positivity as a basis for reusing EV or selecting another Nectin-4-targeted treatment. To address that question, expression at progression needs to be linked to subsequent treatment outcomes.

### 3.5 HER3

The HERTHENA-Lung01 study of patritumab deruxtecan (HER3-DXd) involved EGFR-mutant lung cancer, whereas ICARUS-BREAST01 enrolled patients with HR-positive/HER2-negative breast cancer (36, 77). Existing tissue studies mainly examine the relationships among HER3 expression and spatial distribution, intratumoral drug distribution, and treatment response. Independent studies have obtained paired tissue samples early during HER3-DXd treatment. Krop et al. analyzed 15 pairs of pretreatment biopsies and biopsies obtained on cycle 2, day 3 or cycle 3, day 3 and found a decrease in the proportion of tumor cells with positive HER3 membrane staining. Some patients continued to benefit from treatment after expression decreased (20). TOT-HER3 Part A obtained 54 pairs of specimens for HER3 IHC before a single dose and 21 ± 3 days later. The median change was −5 percentage points, and RNA analyses also identified changes in proliferation- and immune-related genes (21). These results show that an early antigen decrease can accompany a treatment response, adding to the understanding of target changes at different treatment stages.

ICARUS-BREAST01 collected tissue at baseline, during treatment, and at the end of treatment. On-treatment biopsies were scheduled for cycle 1, day 3; cycle 1, day 19; or cycle 2, day 3 (36). Analysis of 39 pairs of baseline and on-treatment imaging mass cytometry (IMC) samples showed a greater decrease in the proportion of HER3-positive cells in responders after 1–2 doses. The study also performed spatial analyses of 11 progression specimens using diaminobenzidine-based IHC (DAB-IHC) and hematoxylin and eosin (H&E) staining to evaluate the spatial distribution of HER3-positive cells and surrounding tissue (36). These 11 progression specimens differed from the 39 early IMC pairs in sampling time and analytical method and should be considered separately. These tissue studies documented the spatial distribution of HER3 and the drug and found that some early changes were associated with response.

Yu et al. used blood testing to examine genetic changes at progression after HER3-DXd treatment and identified acquired ERBB3 and TOP1 alterations in patients’ circulating tumor DNA (ctDNA) (78). In functional models, experiments involving ERBB3 Y789fs showed reduced HER3 expression; in a frameshift model at a nearby site, free payload in the culture medium was also reduced after HER3-DXd exposure. The genetic alterations in patient blood and the protein changes in models provide molecular and functional clues, respectively, that complement the tissue expression studies described above.

The post-treatment HER3 findings show that the meaning of an antigen decrease depends on the timing of assessment. An early decrease can accompany response, whereas specimens collected at progression are used to investigate changes at resistance (20, 21, 36, 78). An early decrease in HER3 alone therefore does not establish treatment failure or justify stopping treatment. HER3 retesting is currently used mainly to study drug activity and treatment response; no expression cutoff has been established for selecting a subsequent ADC.

### 3.6 Current evidence and research needs for CLDN18.2

Clinical studies of CLDN18.2-targeted drugs have used different expression requirements. The randomized GLOW study of the unconjugated antibody zolbetuximab required high expression (79), whereas the testing criteria used for CLDN18.2 ADCs are not identical. The early-phase KYM901 study of CMG901 (also known as AZD0901) evaluated the drug in advanced gastric and gastroesophageal junction cancers, with respective eligibility criteria for its dose-escalation and expansion phases (80). These eligibility criteria apply to the respective drug, study phase, and population, and these differences should be retained when comparing results. The phase I expansion cohort of IBI343 required membrane staining of at least 1+ in at least 1% of tumor cells and further defined a high-expression subgroup as at least 75% of cells with 2+/3+ staining (81). The first cutoff was used for enrollment and the second for subgroup analysis; they served different purposes. The expression criteria used by different drugs and trials must therefore be interpreted in the context of their respective protocols.

Paired patient samples have demonstrated differences in CLDN18.2 between primary and metastatic lesions. Choi et al. analyzed 135 patients with metastatic gastric cancer who had paired primary and metastatic samples; 34 patients had discordant positivity classifications (82). Another study observed reduced CLDN18.2 expression after zolbetuximab plus chemotherapy (83). Direct protein data after ADC treatment have been reported in the preliminary conference report of CLARITY-PT-01, which evaluated CMG901 (AZD0901; sonesitatug vedotin). Among 15 evaluable pairs of baseline and cycle 2 IHC specimens, CLDN18.2 expression decreased in 6, was unchanged in 7, increased in 1, and was negative at both time points in 1 (22). This report shows that expression can change in different directions early during treatment. Follow-up is needed to determine the clinical meaning of these changes; a decrease in cycle 2 should not be equated with resistance.

The cited CLARITY-Gastric01 protocol excludes patients previously treated with an ADC carrying a monomethyl auristatin E (MMAE) payload or with CLDN18.2-targeted therapy other than unconjugated monoclonal antibodies; G-HOPE-001 excludes patients previously treated with an ADC carrying a topoisomerase inhibitor payload (35, 84). Thus, even at the same CLDN18.2 expression level, eligibility for these trials also depends on prior exposure to targeted drugs and payloads. For CLDN18.2, retesting can first establish expression in the currently sampled lesion and determine whether it meets the expression requirement of the intended study protocol. Early paired findings add information on target status during treatment; the relationship between these changes and the efficacy of the next CLDN18.2-targeted ADC has yet to be established (22). Meeting a study’s expression threshold and benefiting after prior ADC treatment therefore need to be evaluated separately.

The patient specimen studies summarized in **Table 1** show that target antigen changes after ADC treatment vary across targets. HER2 and FRα decreased in some specimens collected at progression, suggesting that previous expression results may no longer represent the current lesion (17, 42, 43). TROP2 and Nectin-4 were retained, or even increased, in some resistant specimens, showing that progression can occur while the target remains present (5, 18, 71, 74). HER3 studies showed that an early decrease in expression can accompany treatment response, giving it a different meaning from a decrease at progression (20, 21, 36). Paired CLDN18.2 data currently mainly describe early changes, providing a starting point for following antigen status through progression and before subsequent treatment (22). Together, these studies show how retesting documents the actual antigen state after treatment and helps relate expression changes to the response at that time. Comparisons across targets should first focus on these patterns. Comparisons of the proportions with decreased expression require studies with similar patient populations, sampling schedules, and testing methods.

## 4 Discussion and future directions

### 4.1 Biological significance and clinical uses of reassessment

Taken together, the evidence shows that a pretreatment measurement may no longer represent a tumor’s antigen status, and that treatment failure is not always accompanied by antigen loss. Post-treatment reassessment distinguishes these situations, helping to investigate resistance and check whether drug-specific expression requirements are still met. Its clinical value also depends on whether updated antigen information changes subsequent treatment choices and improves patient outcomes. Decreases in HER2 or FRα after ADC treatment raise the question of whether the current lesion still meets the expression requirement for the intended drug (17, 42, 43). For FRα, for example, a decrease in H-score after progression on MIRV indicates reduced antigen expression, whereas PS2+ measures the proportion of cells reaching a specified membrane staining intensity (17, 33). These measures are not interchangeable. Whether a specimen has crossed the expression threshold for a subsequent treatment must be determined using that drug’s criteria. Reporting a decrease in expression and reclassifying a specimen under treatment criteria therefore provide different information: one describes the antigen change, and the other establishes whether the current specimen still meets the expression requirement. Reporting both would help clarify whether antigen changes affect eligibility for a trial or treatment.

Current uses of TROP2- and Nectin-4-targeted ADCs do not specify routine target antigen IHC cutoffs for patient selection (31, 32, 66), so post-treatment testing is currently used more often to investigate resistance. Retained expression establishes that the antigen is still detectable at progression; effective drug uptake and payload activity are separate questions. Functional studies of impaired EV uptake and cross-resistance to TOP1 inhibitor payloads provide specific explanations for resistance at these steps (23, 30). Patient specimens document the post-treatment state, and functional experiments test whether a particular change weakens drug activity. Together, they help explain why antigen-positive lesions can remain resistant and why studies of subsequent ADCs need to consider both the target and the payload. Continuing a similar drug solely because IHC remains positive would leave these potential causes of resistance unexamined.

Clinical evidence also shows that the use of retesting depends on the treatment setting. In gastric cancer, reconfirmation of HER2 after progression on trastuzumab has been used to select patients for their first T-DXd treatment (51, 52). In the exploratory KATHERINE analysis, patients whose residual tumors became HER2-negative could still benefit from T-DM1 in a specific adjuvant setting (49). These findings after non-ADC treatment illustrate both the practical use of reconfirming expression requirements and the possibility of benefit despite loss of HER2 positivity. After progression on an ADC, whether the current result meets an expression requirement should be evaluated separately from whether using that result to choose the next drug is effective. The former can be assessed against specific testing criteria. The latter requires linking retest results, actual treatment choices, and patient outcomes to determine whether retesting adds useful information beyond treatment history and the evidence already available for drug selection.

### 4.2 Antigen recovery and subsequent ADC treatment

The studies reviewed here have improved our understanding of antigen status at particular time points after treatment. Less is known about how long these changes persist and how they affect the next treatment. Following antigen status through the course of treatment would help establish whether expression recovers after treatment stops and whether patients respond again when the same ADC is reused or a different ADC is given. In withdrawal models, some cell states induced by SG could revert (85), whereas T-DM1-resistant cells could remain resistant long after drug withdrawal (86). An anaplastic large cell lymphoma model of the CD30-targeted ADC brentuximab vedotin (BV) illustrates the relationship more directly. After 26 weeks without the drug, CD30 expression partially recovered, yet the cells remained approximately 11-fold more resistant to BV than the parental cells (87). Recovery of antigen expression therefore does not necessarily coincide with recovery of drug sensitivity. Serial patient data after treatment withdrawal are limited for the six solid-tumor targets reviewed here, and the time required for recovery needs to be determined in patient specimens. Recording both antigen changes and the response to retreatment is necessary to establish whether recovery is accompanied by renewed clinical benefit.

Studies of sequential therapy also need to distinguish a change in target from a change in payload. T-DXd after T-DM1 retains HER2 as the target and changes the payload class, and its efficacy has been demonstrated in a randomized trial (46). Sequencing SG and T-DXd changes the target while retaining TOP1 inhibitor payloads (47, 48). Together with models of payload-related cross-resistance (30), these findings raise specific questions for further study: whether changing the payload improves response when the antigen is retained, and whether pre-existing payload resistance still affects the next ADC when antigen expression decreases and a different target is selected. Clinical data on SG after EV also show variation in treatment response despite changes in both target and payload (88). These findings support studying retest results together with the characteristics of the subsequent drug, rather than choosing a treatment sequence from the direction of antigen change alone.

In summary, research on target antigen reassessment after ADC treatment still consists mainly of patient specimen studies and investigations of resistance mechanisms. These studies have documented antigen changes in post-treatment lesions and identified several reasons why resistance can develop despite target retention. Current specimens can be assessed against the expression requirements of a treatment and, when combined with functional experiments, help explain resistance. The next step is to establish, within specific cancer types and ADC treatment sequences, whether this information helps select more effective drugs and improves patient outcomes.
